# Coupled Dynamics of Information–Epidemic Spreading with Resource Allocation and Transmission on Multi-Layer Networks

**DOI:** 10.3390/e27101080

**Published:** 2025-10-19

**Authors:** Qian Yin, Zhishuang Wang, Kaiyao Wang, Zhiyong Hong

**Affiliations:** School of Electronics and Information Engineering, Wuyi University, Jiangmen 529020, China

**Keywords:** multilayer networks, information diffusion, resource transmission, disease spreading

## Abstract

The spread of epidemic-associated panic information through online social platforms, as well as the allocation and utilization of therapeutic defensive resources in reality, directly influences the transmission of infectious diseases. Moreover, how to reasonably allocate resources to effectively suppress epidemic spread remains a problem that requires further investigation. To address this, we construct a coupled three-layer network framework to explore the complex co-evolutionary mechanisms among false panic information, therapeutic defensive resource transmission, and disease propagation. In the model, individuals can obtain therapeutic defensive resources either through centralized distribution by government agencies or through interpersonal assistance, while the presence of false panic information reduces the willingness of neighbors to share resources. Using the microscopic Markov chain approach, we formulate the dynamical equations of the system and analyze the epidemic threshold. Furthermore, systematic simulation analyses are carried out to evaluate how panic information, resource-sharing willingness, centralized distribution strategies, and resource effectiveness affect epidemic prevalence and threshold levels. For example, under a representative parameter setting, the infection prevalence decreases from 0.18 under the random allocation strategy to 0.03 when resources are allocated exclusively to infected individuals. Moreover, increasing the total supply of resources under high treatment efficiency raises the epidemic threshold by approximately 2.5 times, effectively delaying the outbreak. These quantitative results highlight the significant role of allocation strategies, resource supply, and treatment efficiency in suppressing epidemic transmission.

## 1. Introduction

The sudden emergence and rapid spread of novel infectious diseases can exert profound and far-reaching impacts on society [1]. For instance, the emergence of SARS [2] in 2003 and the global spread of COVID-19 [3] since 2019 brought serious challenges to healthcare systems, disrupted economic activities, undermined social stability, and negatively affected global well-being. Understanding the propagation dynamics of such diseases is therefore critical for elucidating their transmission mechanisms, forecasting epidemic trajectories, and devising effective containment strategies [4,5]. Unlike endemic infectious diseases with well-characterized patterns, emerging epidemics are strongly shaped by a variety of external factors [6]. Elements such as public anxiety, information dissemination, and the allocation and availability of preventive and therapeutic resources significantly influence the course of epidemics [7,8,9,10]. Consequently, a comprehensive investigation of their transmission dynamics must account for these external influences and examine their co-evolutionary interactions with disease spread.

In traditional studies of spreading dynamics, the modeling process often simplifies contact relationships by assuming that individuals in a population are homogeneously mixed [11,12,13]. While this assumption facilitates mathematical analysis, it neglects the inherent heterogeneity of real-world contact patterns [14,15]. Complex networks, as a powerful framework for capturing such heterogeneity, have been widely applied to the analysis of spreading processes, substantially enriching both the theoretical foundations and analytical tools of the field [16,17,18,19,20,21]. However, in reality, spreading processes seldom occur in isolation; instead, they often interact and co-evolve [22,23,24]. For example, during an infectious disease outbreak, online information dissemination can either amplify or mitigate public panic, thereby influencing individuals’ contact behaviors in physical networks and ultimately reshaping the trajectory of disease transmission [25,26,27]. Single-layer network models fall short in representing the diversity of contact patterns across different processes and in capturing their intricate interaction mechanisms. To overcome these limitations, researchers have introduced the concept of multilayer complex networks. In this framework, distinct network layers represent different spreading processes, while inter-layer connections describe their dependencies, enabling a more comprehensive and accurate representation of real-world coupled spreading phenomena [28,29,30]. As a result, multilayer complex networks have become an important and widely adopted tool for exploring the co-evolution of propagation processes.

In recent years, the study of coupled spreading dynamics through multilayer complex networks has swiftly emerged as a focal point in academic research [31,32,33], especially as risk perception induced by information dissemination has attracted extensive attention in the study of epidemic dynamics [34,35,36]. Numerous investigations have centered on the interplay between message diffusion and contagion transmission within two-layer network frameworks [37,38,39,40,41,42]. For example, Massaro and Bagnoli investigated the interplay between epidemic spreading and risk perception within a multilayer network framework, and proposed a self-organized percolation method to characterize how the difference between information and physical contact networks affects the epidemic threshold, revealing the mechanism by which epidemics become difficult to suppress when the two networks differ significantly [43]. Gao et al. incorporated a composite metapopulation model into the disease-spreading layer, examining how the spread of information and the mobility of populations influence disease dynamics [44]. Their results suggest that enhancing information dissemination proves more effective in controlling disease spread than merely reducing individual mobility. Similarly, Feng et al. employed signed networks to model friendly and antagonistic interactions within social structures, exploring how the diversity of social connections and the adaptive modification of preventive actions affect disease transmission on two-layer complex networks [45]. Their analysis reveals that friendly relationships within social networks contribute to suppressing disease spread, whereas fluctuations in preventive measures do not significantly alter the outbreak threshold. Furthermore, An et al. expanded on this by considering the role of medical resources in influencing individual mobility within a dual-layer metapopulation-based scheme for analyzing the co-evolution of awareness diffusion and epidemic processes [46]. They found that uneven distribution of medical resources can lead to population clustering in resource-rich areas, thereby impacting the overall characteristics of disease transmission.

Some researchers have further extended the study of coupled spreading dynamics to three-layer complex networks, investigating the interactions between infectious diseases and additional influencing factors. For instance, Ye et al. developed a heterogeneous framework integrating disease dynamics, behavioral factors, and information spread, finding that information dissemination is essential for controlling epidemic amplification, while behavior can directly reduce the scale of outbreaks [47]. Zhu et al. introduced three-layer networks to analyze how resource allocation driven by information influences the propagation characteristics of epidemic with a latency period. Their findings revealed that highly effective resources can induce a discontinuous-to-continuous phase transition in epidemic spread characteristics [48]. In their study of the co-diffusion of detrimental information, behavior, and disease, Huo and Yu considered factors such as individual self-recognition and physical fitness, concluding that slowing the spread of negative information, enhancing individual cognition, and improving physical fitness all contribute to more effective epidemic control [49]. Additionally, Han and Wang explored the coupled spreading dynamics of beneficial and adverse information alongside infectious diseases affected by two opposing mass communication channels within a three-layer complex network [50]. They discovered that encouraging the circulation of unfavorable news about the outbreak through mass media is more beneficial for epidemic control than promoting the spread of positive information.

Although considerable progress has been made in studying coupled spreading dynamics on multilayer complex networks, several crucial aspects remain underexplored. In particular, the interaction between panic information dissemination and shortages of therapeutic resources—often triggered by outbreaks of novel infectious diseases—has not been thoroughly examined in terms of its impact on disease transmission dynamics. In this study, we focus exclusively on the dissemination of panic-related false information, which plays a central role in triggering resource hoarding behaviors and influencing epidemic transmission dynamics. Panic-driven information spread may prompt individuals to hoard therapeutic resources, reducing mutual aid behaviors and thereby aggravating resource scarcity. This not only amplifies public anxiety but can also create a self-reinforcing cycle of information dissemination and resource hoarding. Persistent inequality and scarcity in resource distribution may further undermine the effectiveness of preventive measures, ultimately weakening collective defense capacity. While panic information primarily propagates via social media, mutual aid in therapeutic resources depends on social networks, and infectious diseases spread through physical contact or airborne droplets. The distinct pathways and mechanisms of these three processes suggest that a three-layer complex network framework can more accurately capture their individual dynamics and mutual influences. Therefore, integrating panic information spread, therapeutic resource distribution and mutual aid, and epidemic transmission into a coupled spreading model on three-layer complex networks is essential for more precise simulation and prediction of epidemic dynamics.

In this study, we investigate the co-evolutionary processes of panic information, therapeutic resources, and infectious diseases under a tri-layer complex network framework. The first layer captures the dissemination of panic information, the second models the distribution of therapeutic resources and mutual assistance behaviors, and the third represents the spread of contagious illnesses. In the proposed model, individuals can obtain therapeutic resources through centralized social distribution or neighborly mutual aid. Infected individuals who acquire these resources have an increased probability of recovery; however, the spread of panic information can diminish the willingness to share resources. Applying the microscopic Markov chain (MMC) approach, we construct the dynamic equations governing various system states in the co-evolutionary model and determine the epidemic threshold on the basis of steady-state analysis. Systematic simulations corroborate the correctness of the formulated equations and the derived epidemic threshold. Furthermore, our numerical experiments examine the effects of panic information dissemination intensity, resource quantity, distribution strategies, and treatment efficacy on epidemic spread, revealing the complex interplay of these factors in shaping overall dynamics. These findings offer theoretical insights for interpreting and managing the diffusion of emerging contagious diseases.

The structure of this paper is arranged as follows. Section 2 introduces the co-evolutionary spreading framework within the three-layer complex network framework setting. Section 3 derives the system equations governing the co-evolutionary processes and presents the theoretical assessment. Section 4 examines the dynamical characteristics of the proposed framework using simulation results. Finally, Section 5 highlights the main outcomes of this study.

## 2. Co-Evolutionary Spreading Model

**Figure 1:**
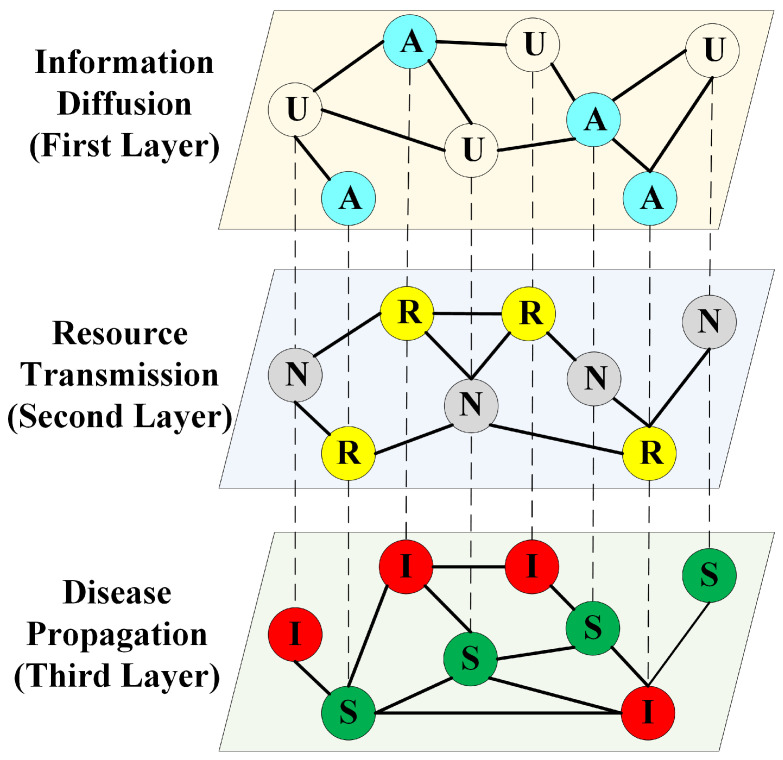
Tri-layer interconnected network framework. The first stratum illustrates information diffusion, the second represents resource transmission, and the third captures epidemic propagation. Within each layer, links denote the connections between nodes, while inter-layer dashed lines indicate that the same node participates in multiple spreading processes across different layers.

To investigate the co-evolutionary spreading relationships among information, resources, and diseases in real-world scenarios, we employ complex network theory to abstract these processes into a three-layer coupled system model, as illustrated in Figure 1. The first layer denotes an online social platform (e.g., Twitter, Weibo) and describes the diffusion of false panic information. Each node in this layer can exist in two possible states: non-spreading (U) or actively transmitting panic information (A). The second and third layers correspond to real-world physical contact networks (e.g., family, friends), which, respectively, characterize the transfer of therapeutic resources and the propagation of contagious diseases. In the second layer, each node has two possible states: without resources (N) or with resources (R). In the third layer, nodes are classified as susceptible (S) or infected (I). When an epidemic spreads through the physical contact network, it can trigger the circulation of panic messages in the online social platform. The spread of such information, in turn, influences resource transfer in the physical contact network, which subsequently affects epidemic transmission. Together, these processes form a coupled co-evolutionary system. All three layers are considered to incorporate the same set of nodes, forming a layer-to-layer one-to-one correspondence. Each dashed line connecting two nodes indicates that they represent the same individual engaged in multiple spreading processes. We assume that every individual simultaneously participates in panic information dissemination, resource transfer, and disease propagation. The following subsections provide a detailed description of these three processes.

### 2.1. Information Diffusion

**Figure 2:**
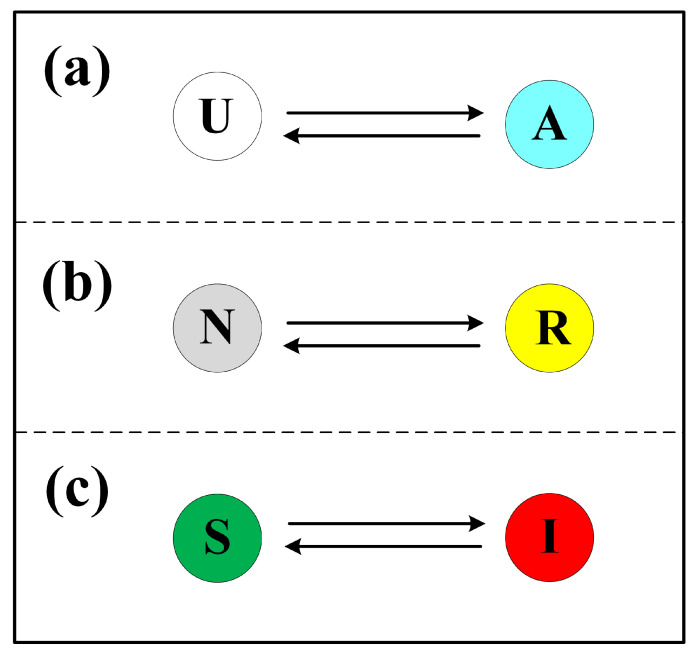
State transitions in the co-evolutionary spreading model. (**a**) State transitions in the diffusion of false panic information; (**b**) state transitions in the process of resource transmission; (**c**) state transitions in the process of epidemic spreading.

To characterize the spread of false panic information, we adopt the UAU model, in which nodes are classified into two distinct states: U (unaware or non-spreading) or A (aware and actively spreading). Nodes in state U refrain from transmitting false panic messages and will attempt to transfer resources to one of their infected neighbors. Similarly, individuals in state A spread false panic information while also transferring resources to one of their infected neighbors. However, unlike individuals in state U, those in state A exhibit a reduced probability of transferring resources. The transition mechanism of the UAU model resembles that of the classical SIS (Susceptible–Infected–Susceptible) model, in which nodes shift between states U and A according to certain probabilities. Specifically, a node in state U becomes state A with probability λ, while a node in state A reverts to state U with probability δ. As illustrated in Figure 2a, these transitions define the dynamics between states U and A.

In addition, considering the coupling effects across network layers, if an individual is in state N (without resources) in the second-layer network and simultaneously in state I (infected) in the third-layer network—meaning the individual is infected but lacks therapeutic resources to facilitate recovery—then the node in the first-layer network transitions from state A to state U. This is because, without access to recovery-promoting resources, the individual must request aid from neighbors who possess resources and will thus cease the dissemination of false panic information.

### 2.2. Resource Transmission

The transfer of resources is described by the NRN process (No Resource–Resource–No Resource), as illustrated in Figure 2b, which shows the state transitions during resource transfer. Here, resources mainly refer to therapeutic resources required for the recovery of infected individuals (hereafter simply referred to as resources). Individuals can obtain resources through two channels: (1) centralized distribution by external sources (e.g., government or medical institutions); (2) transfer from healthy individuals with resources to their infected neighbors without resources.

Specifically, for centralized distribution, external sources allocate ηN resources to individuals in the network at each time step, either randomly or to designated nodes, where 0≤η≤1 represents the proportion of distributed resources relative to the total population *N*. Under the random allocation strategy, both susceptible and infected individuals may obtain resources. Once an infected individual receives resources, they are immediately used to promote recovery and are completely consumed within one time step.

For interpersonal transfer, susceptible individuals with resources may transfer them entirely to one randomly selected infected neighbor without resources, or alternatively, choose not to transfer and keep the resources for potential future use in case of infection. After completing a transfer, the donor no longer retains the resources. Importantly, if no infected neighbor requests resources, healthy individuals will not proactively initiate a transfer. Moreover, considering the inhibitory effect of panic information on resource transfer in the first-layer network, individuals in state U transfer resources to an infected neighbor with probability $\alpha_{i}$, whereas those in state A do so with likelihood $\theta \alpha_{i}$, where 0≤θ≤1 serves as a mitigation parameter quantifying the inhibitory impact of panic information. In particular, when θ=0, individuals in state A completely refuse to transfer resources to infected neighbors; when θ=1, resource transfer is unaffected by panic information.

### 2.3. Disease Propagation

The epidemic propagation process is modeled using the classical SIS process, as illustrated in Figure 2c. In this framework, a susceptible node contracts infection from one of its contagious neighbors with probability β. Considering that resources influence the recovery of infected individuals, once an infected node acquires resources through the transfer process, its recovery rate increases. Specifically, the model specifies that infected individuals without access to resources recover at a rate $\mu^{N}=\mu$, whereas those provided with resources recover at $\mu^{R}=\min\left\{\gamma \mu ,1\right\}$, where γ≥1 is the recovery enhancement factor. When γ=1, resources do not improve the recovery rate of infected individuals, meaning that resource transfer in the second-layer network has no impact on epidemic dynamics within the third-layer network.

Due to the interdependence among the three layers of the coupled network, cross-layer effects are also considered. If an infected individual does not possess resources, in order to improve its probability of recovery, it will request resources from neighbors who hold resources in the second-layer structure. Meanwhile, in the first-layer network, the state of this individual changes to that of a non-spreader of false panic information.

## 3. Theoretical Analysis

In the following, the MMC approach is employed to formulate the dynamical equations of the co-evolutionary system encompassing false panic information, resources, and infectious diseases, and to investigate its epidemic threshold.

### 3.1. Dynamical Equations

**Figure 3:**
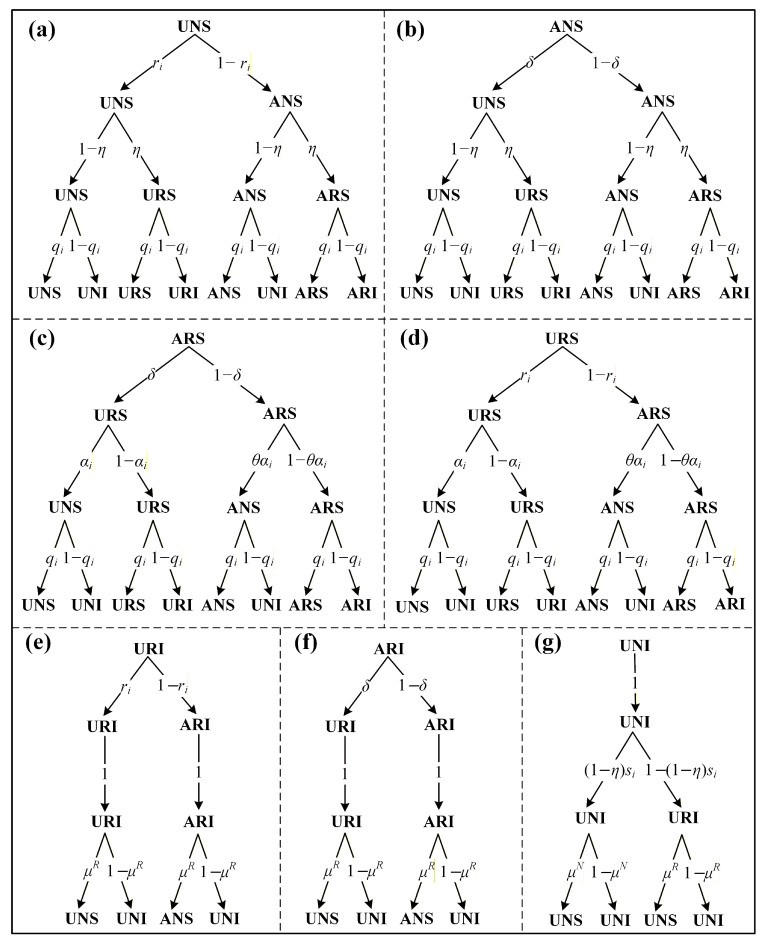
Probability transition trees in the co-evolutionary spreading model. (**a**) The root node is in state UNS; (**b**) the root node is in state ANS; (**c**) the root node is in state ARS; (**d**) the root node is in state URS; (**e**) the root node is in state URI; (**f**) the root node is in state ARI; (**g**) the root node is in state UNI. Each tree illustrates the probability transitions from the root node to the leaf nodes, where the likelihood of a transition is determined by the multiplication of the relevant probabilities along the path from the root to a leaf.

According to the preceding discussion, an individual in the model can occupy one of seven possible configurations: (a) Unaware, without resources, and susceptible (UNS); (b) Aware, without resources, and susceptible (ANS); (c) Aware, with resources, and susceptible (ARS); (d) Unaware, with resources, and susceptible (URS); (e) Unaware, with resources, and infected (URI); (f) Aware, with resources, and infected (ARI); (g) Unaware, without resources, and infected (UNI). It is important to note that if an individual is infected but lacks resources, they must request therapeutic resources from their neighbors to facilitate recovery. In such a case, the individual will cease spreading false panic information, meaning their state in the information diffusion layer will switch from A to U. Consequently, an individual in the state ANI (aware, without resources, and infected) will directly transition to UNI. Therefore, the ANI state is excluded from the model. As illustrated in Figure 3, the transition probabilities among these seven states are represented by seven probability trees, which together capture the full range of possible state transitions in the co-evolutionary spreading process.

${\left[a_{ij}\right]}_{N\times N}$, ${\left[b_{ij}\right]}_{N\times N}$, and ${\left[c_{ij}\right]}_{N\times N}$ are defined as the adjacency matrices corresponding to the first, second, and third network layers, with *N* indicating the number of nodes in each layer. At a given time *t*, every node *i* may occupy one of seven configurations, with the probabilities denoted as $p_{i}^{UNS}\left(t\right),\;p_{i}^{ANS}\left(t\right),\;p_{i}^{ARS}\left(t\right),\;p_{i}^{URS}\left(t\right),\;p_{i}^{URI}\left(t\right),\;p_{i}^{ARI}\left(t\right),$$\;p_{i}^{UNI}\left(t\right).$

Suppose a susceptible individual with resources has no UNI neighbors, then this individual will not transfer resources. If exactly one UNI neighbor exists, the individual will transfer resources to this neighbor with probability ω. Furthermore, for a node in state *U*, at time *t* it transmits resources to one of its infected neighbors with probability $\alpha_{i}\left(t\right)$, defined as follows:

(1) $$\alpha_{i}\left(t\right)=1-{\left(1-\omega \right)}^{\sum_{j}b_{ji}p_{j}^{UNI}\left(t\right)}.$$

From Equation (1), it follows that when $\sum_{j}b_{ji}p_{j}^{UNI}\left(t\right)=0$, we obtain $\alpha_{i}\left(t\right)=0$; and when $\sum_{j}b_{ji}p_{j}^{UNI}\left(t\right)=1$, then $\alpha_{i}\left(t\right)=\omega$.

For an individual in state UNI (infected but lacking resources for recovery), it will request resources from its neighbors that are in states URS or ARS. The probability that a UNI individual *i* obtains resources from its neighbor *j* can be written as

(2) $$\begin{array}{cc} \pi_{ij}\left(t\right) & =\left(\frac{p_{j}^{URS}\left(t\right)\;p_{i}^{UNI}\left(t\right)\;\alpha_{i}\left(t\right)}{\sum_{k}b_{jk}p_{k}^{UNI}\left(t\right)}+\frac{p_{j}^{ARS}\left(t\right)\;p_{i}^{UNI}\left(t\right)\;\theta \alpha_{i}\left(t\right)}{\sum_{k}b_{jk}p_{k}^{UNI}\left(t\right)}\right)\\ \; & =\frac{\left(p_{j}^{URS}\left(t\right)+\theta p_{j}^{ARS}\left(t\right)\right)\;p_{i}^{UNI}\left(t\right)\;\alpha_{i}\left(t\right)}{\sum_{k}b_{jk}p_{k}^{UNI}\left(t\right)}, \end{array}$$

where *k* denotes the neighbors of individual *j*. In particular, if $\sum_{k}b_{jk}p_{k}^{UNI}\left(t\right)=0$, then $\pi_{ij}\left(t\right)=0$.

Assume that, in the first-layer network, an individual *i* in the state of not spreading false panic information will not be influenced by any neighbor that spreads such information at the next time step, with probability denoted as $r_{i}\left(t\right)$. For a node in the UNI condition, the probability of not receiving resources from any of its neighbors is denoted as $s_{i}\left(t\right)$. In the third-layer network, the likelihood that a susceptible node *i* remains uninfected despite the presence of contagious neighbors is denoted as $q_{i}\left(t\right)$. These three probabilities can be expressed as follows:

(3) $$\left\{\begin{array}{c} r_{i}\left(t\right)=\prod_{j}\left[1-a_{ji}p_{j}^{A}\left(t\right)\lambda \right]\\ s_{i}\left(t\right)=\prod_{j}\left[1-b_{ji}\pi_{ij}\left(t\right)\right]\\ q_{i}\left(t\right)=\prod_{j}\left[1-c_{ji}p_{j}^{I}\left(t\right)\beta \right] \end{array}\right..$$

Here, $a_{ji}$, $b_{ji}$, and $c_{ji}$ denote the elements of the adjacency matrices of the first-layer, second-layer, and third-layer networks, correspondingly. Moreover, $p_{j}^{A}\left(t\right)=p_{j}^{ANS}\left(t\right)+p_{j}^{ARS}\left(t\right)+p_{j}^{ARI}\left(t\right),\;p_{j}^{I}\left(t\right)=p_{j}^{URI}\left(t\right)+p_{j}^{ARI}\left(t\right)+p_{j}^{UNI}\left(t\right)$.

By integrating the probability transition trees in Figure 3 with Equation (3), and employing the MMC approach, we establish the dynamical equations for the co-evolutionary spreading model that simultaneously incorporates false panic information, resources, and infectious diseases, expressed as follows:

(4) $$\left\{\begin{array}{cc} p_{i}^{UNS}\left(t+1\right) & =p_{i}^{UNS}\left(t\right)r_{i}\left(t\right)\left(1-\eta \right)q_{i}\left(t\right)+p_{i}^{ANS}\left(t\right)\delta \left(1-\eta \right)q_{i}\left(t\right)+p_{i}^{ARI}\left(t\right)\delta \mu^{R}\\ \; & \;+p_{i}^{ARS}\left(t\right)\delta \alpha_{i}\left(t\right)q_{i}\left(t\right)+p_{i}^{URS}\left(t\right)r_{i}\left(t\right)\alpha_{i}\left(t\right)q_{i}\left(t\right)+p_{i}^{URI}\left(t\right)r_{i}\left(t\right)\mu^{R}\\ \; & \;+p_{i}^{UNI}\left(t\right)\left(1-\eta \right)s_{i}\left(t\right)\mu^{N}+p_{i}^{UNI}\left(t\right)\left[1-\left(1-\eta \right)s_{i}\left(t\right)\right]\mu^{R}\\ p_{i}^{ANS}\left(t+1\right) & =p_{i}^{UNS}\left(t\right)\left[1-r_{i}\left(t\right)\right]\left(1-\eta \right)q_{i}\left(t\right)+p_{i}^{ANS}\left(t\right)\left(1-\delta \right)\left(1-\eta \right)q_{i}\left(t\right)\\ \; & \;+p_{i}^{ARS}\left(t\right)\left(1-\delta \right)\theta \alpha_{i}\left(t\right)q_{i}\left(t\right)+p_{i}^{URS}\left(t\right)\left[1-r_{i}\left(t\right)\right]\theta \alpha_{i}\left(t\right)q_{i}\left(t\right)\\ \; & \;+p_{i}^{URI}\left(t\right)\left[1-r_{i}\left(t\right)\right]\mu^{R}+p_{i}^{ARI}\left(t\right)\left(1-\delta \right)\mu^{R}\\ p_{i}^{ARS}\left(t+1\right) & =p_{i}^{UNS}\left(t\right)\left[1-r_{i}\left(t\right)\right]\eta q_{i}\left(t\right)+p_{i}^{URS}\left(t\right)\left[1-r_{i}\left(t\right)\right]\left[1-\theta \alpha_{i}\left(t\right)\right]q_{i}\left(t\right)\\ \; & \;+p_{i}^{ARS}\left(t\right)\left(1-\delta \right)\left[1-\theta \alpha_{i}\left(t\right)\right]q_{i}\left(t\right)+p_{i}^{ANS}\left(t\right)\left(1-\delta \right)\eta q_{i}\left(t\right)\\ p_{i}^{URS}\left(t+1\right) & =p_{i}^{UNS}\left(t\right)r_{i}\left(t\right)\eta q_{i}\left(t\right)+p_{i}^{ANS}\left(t\right)\delta \eta q_{i}\left(t\right)+p_{i}^{ARS}\left(t\right)\delta \left[1-\alpha_{i}\left(t\right)\right]q_{i}\left(t\right)\\ \; & \;+p_{i}^{URS}\left(t\right)r_{i}\left(t\right)\left[1-\alpha_{i}\left(t\right)\right]q_{i}\left(t\right)\\ p_{i}^{URI}\left(t+1\right) & =p_{i}^{UNS}\left(t\right)r_{i}\left(t\right)\eta \left[1-q_{i}\left(t\right)\right]+p_{i}^{URS}\left(t\right)r_{i}\left(t\right)\left[1-\alpha_{i}\left(t\right)\right]\left[1-q_{i}\left(t\right)\right]\\ \; & \;+p_{i}^{ARS}\left(t\right)\delta \left[1-\alpha_{i}\left(t\right)\right]\left[1-q_{i}\left(t\right)\right]+p_{i}^{ANS}\left(t\right)\delta \eta \left[1-q_{i}\left(t\right)\right]\\ p_{i}^{ARI}\left(t+1\right) & =p_{i}^{UNS}\left(t\right)\left[1-r_{i}\left(t\right)\right]\eta \left[1-q_{i}\left(t\right)\right]+p_{i}^{ANS}\left(t\right)\left(1-\delta \right)\eta \left[1-q_{i}\left(t\right)\right]\\ \; & \;+p_{i}^{URS}\left(t\right)\left[1-r_{i}\left(t\right)\right]\left[1-\theta \alpha_{i}\left(t\right)\right]\left[1-q_{i}\left(t\right)\right]\\ \; & \;+p_{i}^{ARS}\left(t\right)\left(1-\delta \right)\left[1-\theta \alpha_{i}\left(t\right)\right]\left[1-q_{i}\left(t\right)\right]\\ p_{i}^{UNI}\left(t+1\right) & =p_{i}^{UNS}\left(t\right)\left(1-\eta \right)\;\left[1-q_{i}\left(t\right)\right]+p_{i}^{ANS}\left(t\right)\left(1-\eta \right)\;\left[1-q_{i}\left(t\right)\right]\\ \; & \;+p_{i}^{ARS}\left(t\right)\;\delta \alpha_{i}\left(t\right)\;\left[1-q_{i}\left(t\right)\right]+p_{i}^{ARS}\left(t\right)\left(1-\delta \right)\theta \alpha_{i}\left(t\right)\;\left[1-q_{i}\left(t\right)\right]\\ \; & \;+p_{i}^{URS}\left(t\right)\;r_{i}\left(t\right)\;\alpha_{i}\left(t\right)\;\left[1-q_{i}\left(t\right)\right]+p_{i}^{UNI}\left(t\right)\left(1-\eta \right)s_{i}\left(t\right)\left(1-\mu^{N}\right)\\ \; & \;+p_{i}^{ARI}\left(t\right)\left(1-\mu^{R}\right)+p_{i}^{URS}\left(t\right)\left[1-r_{i}\left(t\right)\right]\theta \alpha_{i}\left(t\right)\;\left[1-q_{i}\left(t\right)\right]\\ \; & \;+p_{i}^{UNI}\left(t\right)\left[1-\left(1-\eta \right)s_{i}\left(t\right)\right]\left(1-\mu^{R}\right)+p_{i}^{URI}\left(t\right)\left(1-\mu^{R}\right) \end{array}.\right.$$

For every discrete time instant, the probabilities of all seven possible states must sum to one, i.e., $p_{i}^{UNS}\left(t\right)+p_{i}^{ANS}\left(t\right)+p_{i}^{ARS}\left(t\right)+p_{i}^{URS}\left(t\right)+p_{i}^{URI}\left(t\right)+p_{i}^{ARI}\left(t\right)+p_{i}^{UNI}\left(t\right)=1.$

### 3.2. Epidemic Threshold

Subsequently, a detailed investigation is performed into the epidemic threshold of the co-evolutionary spreading framework that integrates information, resources, and epidemics. When the system evolves for a sufficiently long time, the dynamics among the seven states converge toward equilibrium. That is, the likelihood for a node to occupy each of the seven states $p_{i}^{UNS}\left(t\right)$, $p_{i}^{ANS}\left(t\right)$, $p_{i}^{ARS}\left(t\right)$, $p_{i}^{URS}\left(t\right)$, $p_{i}^{URI}\left(t\right)$, $p_{i}^{ARI}\left(t\right)$, and $p_{i}^{UNI}\left(t\right)$ become equal at time steps *t* and t+1. By imposing the condition that state probabilities remain unchanged across consecutive time steps, the following steady-state equations are obtained:

(5) $$\left\{\begin{array}{c} \lim_{t\to \infty }p_{i}^{UNS}\left(t+1\right)=\lim_{t\to \infty }p_{i}^{UNS}\left(t\right)=p_{i}^{UNS}\\ \lim_{t\to \infty }p_{i}^{ANS}\left(t+1\right)=\lim_{t\to \infty }p_{i}^{ANS}\left(t\right)=p_{i}^{ANS}\\ \lim_{t\to \infty }p_{i}^{ARS}\left(t+1\right)=\lim_{t\to \infty }p_{i}^{ARS}\left(t\right)=p_{i}^{ARS}\\ \lim_{t\to \infty }p_{i}^{URS}\left(t+1\right)=\lim_{t\to \infty }p_{i}^{URS}\left(t\right)=p_{i}^{URS}\\ \lim_{t\to \infty }p_{i}^{URI}\left(t+1\right)=\lim_{t\to \infty }p_{i}^{URI}\left(t\right)=p_{i}^{URI}\\ \lim_{t\to \infty }p_{i}^{ARI}\left(t+1\right)=\lim_{t\to \infty }p_{i}^{ARI}\left(t\right)=p_{i}^{ARI}\\ \lim_{t\to \infty }p_{i}^{UNI}\left(t+1\right)=\lim_{t\to \infty }p_{i}^{UNI}\left(t\right)=p_{i}^{UNI} \end{array}\right..$$

When the system reaches a steady state and $\beta \to \beta_{c}$, the proportion of infected nodes within the population $p_{i}^{I}$ approaches zero. Specifically, we denote $p_{i}^{I}=p_{i}^{URI}+p_{i}^{ARI}+p_{i}^{UNI}=\sigma_{i}\ll 1$. By neglecting higher-order terms, the last two probabilities in Equation (3) are approximated in the following manner:

(6) $$\left\{\begin{array}{c} s_{i}\left(t\right)=\prod_{j}\left[1-b_{ji}\pi_{ij}\left(t\right)\right]\approx 1-\sum_{j}b_{ji}\pi_{ij}\\ q_{i}\left(t\right)=\prod_{j}\left[1-c_{ji}p_{j}^{I}\left(t\right)\beta \right]\approx 1-\beta \sum_{j}c_{ji}\sigma_{j} \end{array}\right..$$

By substituting Equations (5) and (6) into the first four equations of Equation (4), and considering that $p_{i}^{I}\ll 1$, we obtain the following approximations:

(7) $$\left\{\begin{array}{c} p_{i}^{UNS}=p_{i}^{UNS}r_{i}\left(1-\eta \right)+p_{i}^{ANS}\delta \left(1-\eta \right)\\ p_{i}^{ANS}=p_{i}^{UNS}\left(1-r_{i}\right)\left(1-\eta \right)+p_{i}^{ANS}\left(1-\delta \right)\left(1-\eta \right)\\ p_{i}^{ARS}=p_{i}^{UNS}\left(1-r_{i}\right)\eta +p_{i}^{URS}\left(1-r_{i}\right)+p_{i}^{ARS}\left(1-\delta \right)+p_{i}^{ANS}\left(1-\delta \right)\eta \\ p_{i}^{URS}=p_{i}^{UNS}r_{i}\eta +p_{i}^{ANS}\delta \eta +p_{i}^{ARS}\delta +p_{i}^{URS}r_{i} \end{array}\right..$$

In a similar manner, the final three relations of Equation (4) can be formulated as follows:

(8) $$\left\{\begin{array}{cc} p_{i}^{URI} & =p_{i}^{UNS}r_{i}\eta \beta \sum_{j}c_{ji}\sigma_{j}+p_{i}^{URS}r_{i}\left(1-\alpha_{i}\right)\beta \sum_{j}c_{ji}\sigma_{j}\\ \; & \;+p_{i}^{ARS}\delta \left(1-\alpha_{i}\right)\beta \sum_{j}c_{ji}\sigma_{j}+p_{i}^{ANS}\delta \eta \beta \sum_{j}c_{ji}\sigma_{j}\\ p_{i}^{ARI} & =p_{i}^{UNS}\left(1-r_{i}\right)\eta \beta \sum_{j}c_{ji}\sigma_{j}+p_{i}^{ANS}\left(1-\delta \right)\eta \beta \sum_{j}c_{ji}\sigma_{j}\\ \; & \;+p_{i}^{URS}\left(1-r_{i}\right)\left(1-\theta \alpha_{i}\right)\beta \sum_{j}c_{ji}\sigma_{j}+p_{i}^{ARS}\left(1-\delta \right)\left(1-\theta \alpha_{i}\right)\beta \sum_{j}c_{ji}\sigma_{j}\\ p_{i}^{UNI} & =p_{i}^{UNS}\left(1-\eta \right)\beta \sum_{j}c_{ji}\sigma_{j}+p_{i}^{ANS}\left(1-\eta \right)\beta \sum_{j}c_{ji}\sigma_{j}\\ \; & \;+p_{i}^{ARS}\delta \alpha_{i}\beta \sum_{j}c_{ji}\sigma_{j}+p_{i}^{ARS}\left(1-\delta \right)\theta \alpha_{i}\beta \sum_{j}c_{ji}\sigma_{j}\\ \; & \;+p_{i}^{URS}r_{i}\alpha_{i}\beta \sum_{j}c_{ji}\sigma_{j}+p_{i}^{URS}\left(1-r_{i}\right)\theta \alpha_{i}\beta \sum_{j}c_{ji}\sigma_{j}\\ \; & \;+p_{i}^{ARI}\left(1-\mu^{R}\right)+p_{i}^{UNI}\left(1-\eta \right)s_{i}\left(1-\mu^{N}\right)\\ \; & \;+p_{i}^{UNI}\left[1-\left(1-\eta \right)s_{i}\right]\left(1-\mu^{R}\right)+p_{i}^{URI}\left(1-\mu^{R}\right) \end{array}.\right.$$

Based on $p_{i}^{I}=p_{i}^{URI}+p_{i}^{ARI}+p_{i}^{UNI}=\sigma_{i}\ll 1$, Equation (8) can be further simplified to obtain

(9) $$\beta p_{i}^{S}\sum_{j}c_{ji}\sigma_{j}=\left[\eta \mu^{R}+\left(1-\eta \right)\mu^{N}\right]\sigma_{i}.$$

Next, as the system approaches equilibrium with $p_{i}^{I}\ll 1$, it follows that $p_{i}^{S}\to 1$. Therefore, Equation (9) can be rewritten as

(10) $$\sum_{j}\left\{c_{ji}-\frac{\eta \mu^{R}+\left(1-\eta \right)\mu^{N}}{\beta }\;\varepsilon_{ji}\right\}\sigma_{j}=0,$$

where $\varepsilon_{ji}$ denotes the elements of the identity matrix.

Therefore, the critical threshold of the information–resource–disease co-evolution spreading model is given by

(11) $$\beta_{c}=\frac{\eta \mu^{R}+\left(1-\eta \right)\mu^{N}}{\Delta_{\max}\left(C\right)},$$

where $\Delta_{\max}\left(C\right)$ corresponds to the principal eigenvalue of the adjacency matrix characterizing the third-layer network.

According to Equation (11), it can be observed that $\beta_{c}$ is closely linked to the fraction of resources distributed within the system, the recovery performance of individuals either possessing or lacking resources, and the structural topology of the third-layer network. In particular, when η=0, meaning that no external resources are allocated to the individuals in the model, the epidemic threshold simplifies to $\beta_{c}=\frac{\mu^{N}}{\Delta_{\max}\left(C\right)}$. When η=1, meaning that all individuals in the system receive external resources, the epidemic threshold becomes $\beta_{c}=\frac{\mu^{R}}{\Delta_{\max}\left(C\right)}$.

## 4. Simulation Results

In the following, numerical experiments are performed to examine the validity of the model’s dynamical equations, the epidemic prevalence, and the threshold condition. For the three-layer network, the size of each layer is set to N=10000, with an average degree of 〈k〉=6. In the experiments, the first-layer network is modeled using a BA (Barabási–Albert) scale-free topology, while the second and third layers are considered under two scenarios: an ER (Erdős–Rényi) random topology and a BA scale-free topology. The outcomes obtained from the MMC approach and MC (Monte Carlo) simulations correspond to the steady state of the system, with the MC simulation outcomes reported as the averages over 20 independent realizations. Let $\rho^{A}$, $\rho^{R}$, and $\rho^{I}$ denote the densities of individuals occupying states *A*, *R*, and *I*, respectively. The initial conditions are specified as $\rho^{A}=\rho^{I}=0.1$ and $\rho^{R}=0$.

### 4.1. Accuracy Analysis of the Dynamical Equations

**Figure 4:**
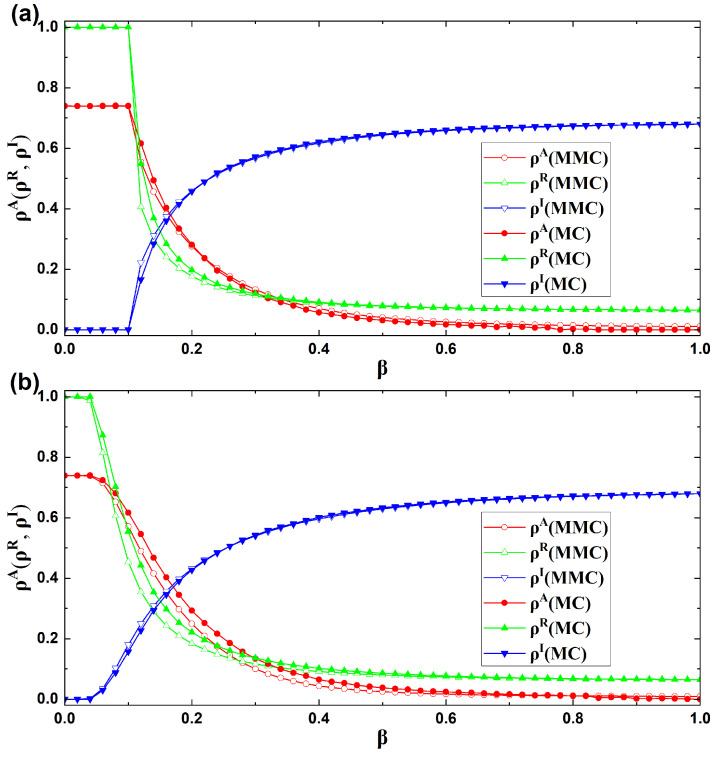
Comparison between MMC and MC results. (**a**) Both the second and third layer networks follow an ER random topology; (**b**) both the second and third layer networks adopt a BA scale-free topology.

The reliability of the dynamical equations is evaluated by contrasting the outcomes from MC simulations with those obtained via the MMC method. As shown in Figure 4, the two approaches are tested under multiple network topologies and infection parameters β, with parameters set to λ=0.5, δ=0.3, η=0.2, ω=0.3, θ=0, μ=0.3, and γ=3. In Figure 4a, when both the second and third layer networks follow an ER random topology, the functions of $\rho^{A}$, $\rho^{R}$, and $\rho^{I}$ with respect to β are shown. In Figure 4b, when both the second and third layer networks adopt a BA scale-free topology, the corresponding functions of $\rho^{A}$, $\rho^{R}$, and $\rho^{I}$ are displayed. Moreover, in both Figure 4a,b, the red lines represent $\rho^{A}$, the green lines represent $\rho^{R}$, and the blue lines represent $\rho^{I}$. Hollow markers correspond to the results obtained from the MMC method, while solid markers denote those obtained from MC simulations. By comparing the MMC and MC results under the same parameters, it becomes clear that the two methods exhibit strong consistency. The simulation results demonstrate that the constructed dynamical equations can accurately capture the co-evolutionary dynamics of panic-related misinformation dissemination, resource transmission, and contagion propagation. To further quantify the accuracy of the dynamical equations, Table 1 reports the average relative error for each data set.

As shown in Table 1, the MMC approach and MC simulations yield $\rho^{R}$ and $\rho^{I}$ with minimal relative errors, suggesting their outcomes are nearly identical. By contrast, the relative error of $\rho^{A}$ is relatively larger. This mainly arises from two aspects. First, when the value of $\rho^{A}$ itself is very small, the denominator in the relative error calculation becomes small, which amplifies the relative error. This does not indicate any substantial discrepancy between the MMC and MC results. For example, in Figure 4a, when β=0.6, the value of $\rho^{A}$ obtained by MMC is 0.0258, while that obtained by MC is 0.0172. The relative error is |0.0258−0.0172|/0.0172=0.5 (i.e., 50%), but the absolute error is only 0.0086. This clearly shows that the two values are actually very close, and the large relative error is purely due to the calculation characteristics. In contrast, when β=0.16 in Figure 4a, the values obtained by MMC and MC are 0.3818 and 0.4027, respectively, with a relative error of approximately 5.19% and an absolute error of 0.0209. Second, another source of error is related to the state transition mechanism of the UAU model under the influence of disease spreading. Specifically, individuals in the UNS state are immediately converted to the ANI state upon infection. The MMC method describes the probability of individuals being in each state, whereas in MC simulations, the state of each individual at each time step is deterministic. This difference between probabilistic and deterministic descriptions leads to slight discrepancies in the actual transition probability from UNS to ANI, resulting in small differences in the steady-state density of state A. Nevertheless, the overall results remain highly consistent, verifying the high accuracy of the constructed dynamical equations in representing the coupled evolution of panic-related misinformation diffusion, resource transmission, and contagion spreading.

By comparing Figure 4a,b, it can be seen that the critical point obtained under different network topologies are not identical, further confirming that the threshold of the model is closely associated with the structural characteristics of the network. Moreover, epidemics are more likely to break out in BA networks than in ER networks. As illustrated in Figure 4, regardless of whether the second and third layer networks adopt an ER random topology or a BA scale-free topology, when the epidemic does not spread (i.e., $\beta <\beta_{c}$), we have $\rho^{I}=0$ and $\rho^{R}=1$, and $\rho^{A}$ is a fixed constant. This indicates that when the disease does not cause large-scale transmission, the system stabilizes with each individual eventually possessing resources. Nevertheless, once β grows beyond the critical point ($\beta >\beta_{c}$), $\rho^{I}$ gradually rises and exhibits a gradual increase, whereas $\rho^{A}$ and $\rho^{R}$ show a corresponding decline. This occurs because a higher infection probability leads to an increasing population of infected nodes in the system. Since the recovery of infected individuals consumes a certain amount of resources, the share of nodes holding resources gradually decreases. Simultaneously, individuals without resources must request them from neighbors who still possess resources. Consequently, they will cease spreading false panic information related to the epidemic. Ultimately, this process leads to a decline in the ratio of individuals disseminating such information within the system.

### 4.2. Analysis of Epidemic Prevalence

**Figure 5:**
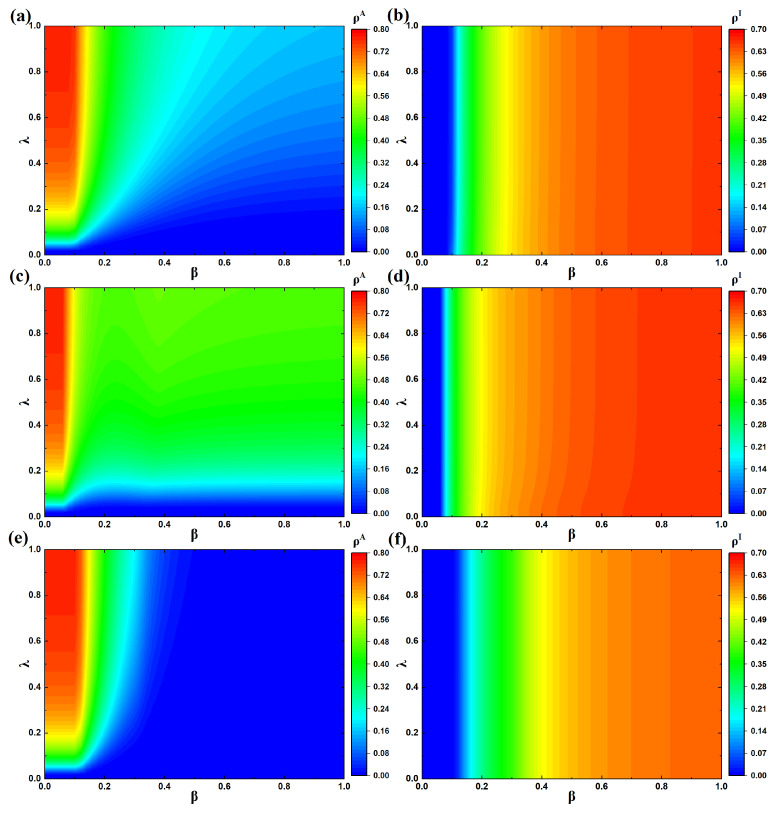
Information and disease prevalence under different resource allocation strategies. (**a**) Variation in $\rho^{A}$ when resources are randomly allocated to all individuals; (**b**) variation in $\rho^{I}$ when resources are randomly allocated to all individuals; (**c**) variation in $\rho^{A}$ when resources are randomly allocated to all susceptible individuals; (**d**) variation in $\rho^{I}$ when resources are randomly allocated to all susceptible individuals; (**e**) variation in $\rho^{A}$ when resources are randomly distributed to all infected individuals; (**f**) variation in $\rho^{I}$ when resources are randomly distributed to all infected individuals.

Next, we conduct a detailed investigation into how the parameters associated with the diffusion of false panic information, resource transmission, and epidemic spreading influence the overall epidemic prevalence. First, we examine the effects of different resource allocation strategies under parameters λ and β on $\rho^{A}$ and $\rho^{I}$. Figure 5 considers the case where both the second and third layer networks are ER random topology, while the remaining conditions are assigned as δ=0.3, η=0.4, ω=0.3, θ=0, μ=0.2, and γ=4. By comparing Figure 5b,d,f, it can be observed that different resource allocation strategies correspond to different epidemic thresholds. The threshold is the smallest in Figure 5d, while it is the largest in Figure 5f. This occurs because in Figure 5d, resources are allocated to susceptible individuals, who may then retain the resources for themselves rather than distributing them to infected neighbors in need, thereby accelerating epidemic spread. By contrast, in Figure 5f, resources are allocated directly to infected individuals, facilitating recovery and thus reducing disease transmission. This suggests that when epidemics spread, targeted allocation of resources is more effective in suppressing epidemic propagation. For example, when β=0.12 and λ=0.32, the infection prevalence under the random allocation strategy and the strategy of allocating resources exclusively to susceptible individuals is 0.18 and 0.37, respectively, while it decreases to only 0.03 under the strategy of allocating resources exclusively to infected individuals, clearly demonstrating the significant differences in epidemic propagation caused by different allocation strategies. By comparing Figure 5a,c,e, it can be observed that during large-scale epidemic outbreaks, the strategy of allocating resources exclusively to infected individuals achieves the strongest suppression effect on the spread of panic-related false information. The underlying mechanism can be further explained based on the probability trees shown in Figure 3. When resources are allocated exclusively to infected individuals, UNS individuals no longer transition to ARS or ARI states. Consequently, the probability that a susceptible individual in state U becomes an information spreader decreases from $\left(1-r_{i}\right)\left(q_{i}+\eta \left(1-q_{i}\right)\right)$ (in the cases of random allocation or allocation to susceptible individuals) to $\left(1-r_{i}\right)q_{i}$, thereby reducing information dissemination to some extent. Similarly, ANS individuals no longer transition to ARS or ARI states, and the probability that a susceptible individual in state A continues to spread panic information decreases from $\left(1-\delta \right)(q_{i}+\eta \left(1-q_{i}\right))$ to $\left(1-\delta \right)q_{i}$, further weakening the information transmission process. In contrast, when resources are randomly allocated or allocated exclusively to susceptible individuals, all states remain in the steady state, and individuals in ARS and ARI states account for a non-negligible proportion of the system, which in turn promotes the spread of panic-related false information under these two allocation strategies.

In addition, Figure 5 illustrates that when the value of λ is fixed, $\rho^{A}$ decreases with increasing β, whereas $\rho^{I}$ increases with β. This is because as the infection probability β grows, the scale of infected individuals in the system expands, and those who have already been infected require resources from their neighbors, thereby ceasing to spread panic-related false information. Consequently, the share of agents transmitting such false message progressively declines. When β is fixed, $\rho^{A}$ increases with λ, while $\rho^{I}$ is only slightly affected. This occurs because as the communication rate λ rises, the count of nodes spreading panic-related false information increases, but the fraction of infected agents in the system stays nearly constant. Moreover, since information transmission primarily influences resource transfer, allocating resources to susceptible individuals makes the change in information transmission rate exert a stronger effect on resource flow, thereby indirectly impacting epidemic prevalence. Therefore, in resource allocation, prioritizing the distribution of therapeutic resources to infected individuals is more effective for controlling epidemic transmission.

**Figure 6:**
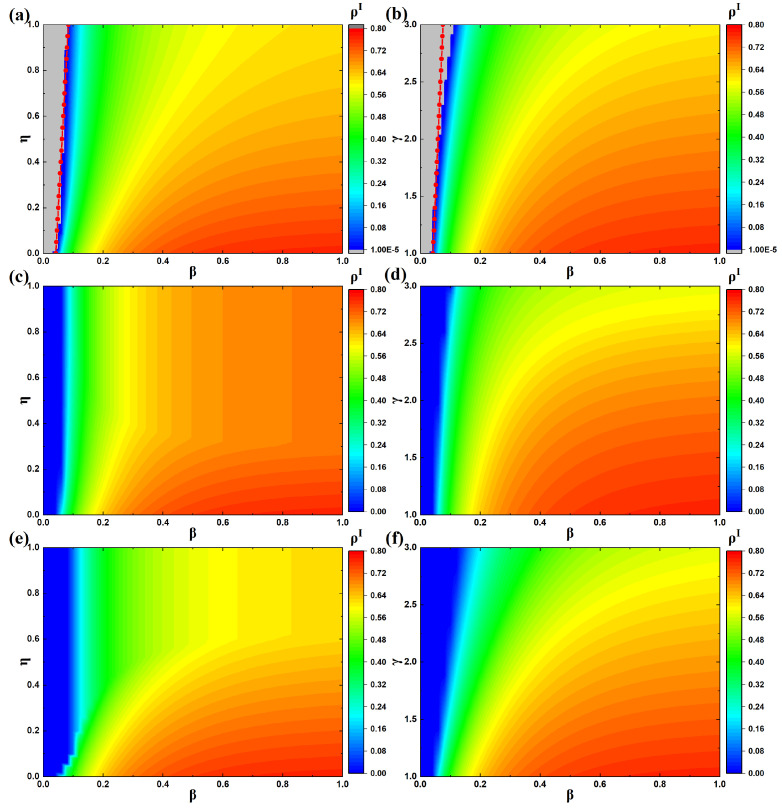
Epidemic prevalence under different parameter settings and resource allocation strategies. (**a**) Effects of β and η on $\rho^{I}$ when resources are randomly allocated to all individuals; (**b**) effects of β and γ on $\rho^{I}$ when resources are randomly allocated to all individuals; (**c**) effects of β and η on $\rho^{I}$ when resources are randomly allocated to all susceptible individuals; (**d**) effects of β and γ on $\rho^{I}$ when resources are randomly allocated to all susceptible individuals; (**e**) effects of β and η on $\rho^{I}$ when resources are randomly allocated to all infected individuals; (**f**) effects of β and γ on $\rho^{I}$ when resources are randomly allocated to all infected individuals.

Next, Figure 6 examines the effects of infection probability β, resource allocation ratio η, and recovery enhancement factor γ on the epidemic prevalence $\rho^{I}$, with λ=0.5, ω=0.3, θ=0, μ=0.3, and δ=0.3 as parameter values. Figure 6a,c,e shows the effects of β and η on $\rho^{I}$, with γ=2, whereas Figure 6b,d,f presents the effects of β and γ on $\rho^{I}$, with η=0.4. In panels (a) and (b), the gray region indicates $\rho^{I}=0$, and the red dashed line represents the epidemic threshold obtained from Equation (11). Comparing panels (a), (c), and (e), one can observe that under different resource allocation strategies, the effects of β and η on $\rho^{I}$ differ. Specifically, in panel (c), the blue shaded area is the smallest, while in panel (e), the blue shaded area is the largest. This means that under the same β and η, allocating resources to all susceptible individuals leads to the largest epidemic outbreak, whereas allocating resources to all infected individuals results in the smallest outbreak. Similarly, comparing panels (b), (d), and (f), a consistent pattern can be found. This further indicates that, for epidemic control, prioritizing the allocation of therapeutic resources to infected individuals proves more efficient in limiting disease transmission.

From Figure 6b,d,f, it is evident that when γ=1, the outbreak threshold is minimized, indicating that even when infected individuals receive resources, their recovery rate does not improve, making epidemic outbreaks more likely. This suggests that the relationship between resource availability and recovery rate directly affects the emergence of infectious diseases. When the illness occurs in the system ($\beta >\beta_{c}$), the prevalence $\rho^{I}$ decreases as γ increases. This phenomenon arises because larger values of γ correspond to higher recovery efficiency for individuals with resources. Thus, when infected individuals obtain resources, they are more likely to recover and revert to the susceptible state, thereby reducing the overall scale of infection. This demonstrates that enhancing the recovery efficiency of infected individuals with resources is beneficial for suppressing epidemic spread. Furthermore, comparing panels (b), (d), and (f), it is evident that when the disease spreads within the system and β is fixed, increasing γ and allocating all resources to infected individuals results in the smallest prevalence of infection. Under the same parameter conditions, panel (f) shows the lightest color for $\rho^{I}$, indicating that allocating resources to infected individuals, while simultaneously enhancing their recovery efficiency, is most effective in mitigating the transmission of illness within the system.

### 4.3. Analysis of the Epidemic Threshold

In this subsection, we investigate how the parameters η and γ shape the epidemic threshold in the co-evolutionary framework that integrates information, resources, and diseases. Figure 6 validates the accuracy of the analytical expression for the epidemic threshold derived from the dynamical analysis (Equation (11)). In Figure 6, the boundary between the blue and gray regions represents the critical threshold that separates outbreak and non-outbreak regimes. When the red dashed curve coincides with this boundary, it confirms that Equation (11) provides an accurate calculation of the threshold. These observations demonstrate strong agreement between the theoretical analysis and the simulation results.

**Figure 7:**
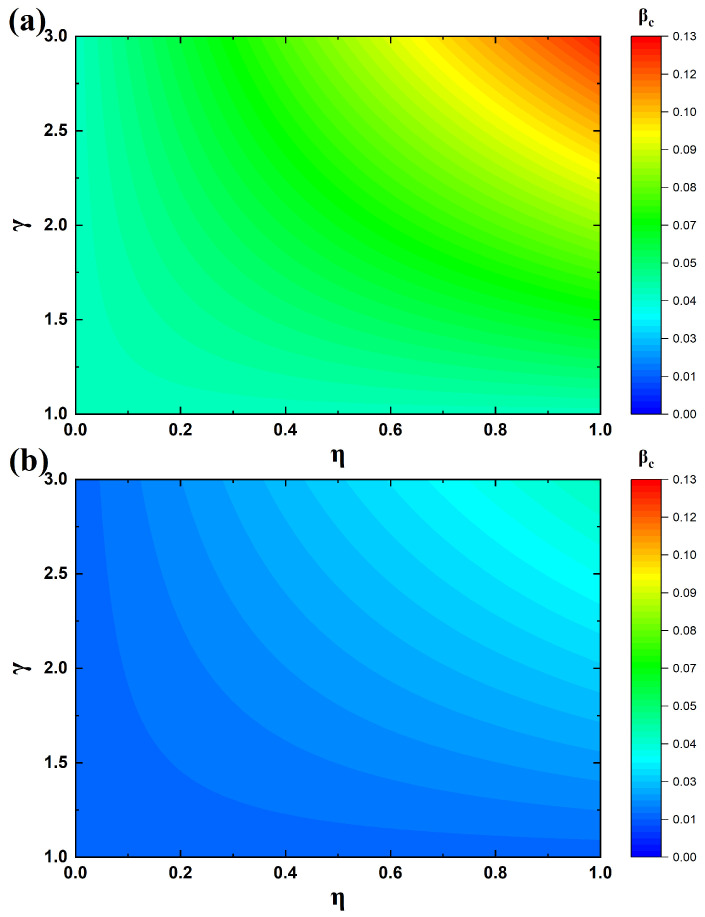
Variation in the epidemic threshold under different parameter settings. (**a**) Both the second and third networks are ER random graphs; (**b**) both the second and third networks are BA scale-free networks.

To give a clearer explanation of how parameters η and γ affect the threshold in the model, Figure 7 illustrates the variations of $\beta_{c}$ under different values of η and γ, with μ=0.3. In Figure 7a, the first layer network is modeled as a BA scale-free topology, while both the second and third layer networks are ER random networks. Figure 7b presents the results when all three layers are BA networks. By comparing Figure 7a,b, it is evident that under the same values of η and γ, the epidemic threshold in the ER networks of panel (a) is significantly larger than that in the BA networks of panel (b), thereby validating the conclusion from Equation (11) in the previous section that the theoretical threshold is strongly associated with network topology. The primary reason for this phenomenon lies in the difference in degree distribution: in ER networks, nodes generally have a relatively uniform degree, whereas BA networks contain hub nodes with substantially higher degrees. Once these hub nodes are infected, the likelihood of further transmission is much greater, as the infection quickly propagates across many other nodes in the network. Therefore, the epidemic threshold is lower in BA networks, making outbreaks more likely compared to ER networks.

Next, we analyze how the epidemic threshold $\beta_{c}$ varies with η and γ. When γ is fixed, $\beta_{c}$ increases as η becomes larger. For example, in Figure 7a, when γ=3, increasing η from 0.1 to 0.9 raises the epidemic threshold from 0.05 to 0.11, corresponding to a 2.5-fold increase. This is because a greater value of η corresponds to more resources being allocated from external sources to individuals, thereby facilitating their recovery and suppressing the outbreak of disease. This result indicates that increasing the amount of resources available to individuals in the system is beneficial for controlling the spread of infection. Moreover, when η is fixed, $\beta_{c}$ increases as γ grows. Similarly, in Figure 7a, when η=0.9, increasing γ from 1 to 3 raises the epidemic threshold from 0.04 to 0.11, corresponding to a 2.75-fold increase. This is due to the fact that a larger γ implies higher recovery rates for individuals with resources, which reduces the share of infected nodes in the population and thus suppresses disease transmission. This demonstrates that improving the recovery efficiency of individuals with resources can effectively control epidemic outbreaks. As shown in Figure 7, regardless of whether the networks are ER networks or BA networks, simultaneously increasing both η and γ yields the largest epidemic threshold $\beta_{c}$. This finding suggests that enhancing the overall resource availability in the system, while simultaneously improving the treatment efficiency of resources, provides a more effective means of controlling epidemic spread.

## 5. Conclusions

In this study, we proposed a novel three-layered network structure to investigate the co-evolutionary dynamics of panic-driven misinformation, therapeutic resources, and infectious diseases. The three interconnected layers, respectively, capture the dissemination of false panic information on virtual social networks, the distribution and transfer of therapeutic resources in social contact networks, and the physical transmission of infectious diseases. By integrating these interacting processes into a unified framework, our model provides a more comprehensive perspective for understanding epidemic dynamics in realistic social systems. We derived the dynamical equations of the co-evolutionary propagation mechanism and rigorously determined the epidemic critical point. The findings indicate that whether an epidemic outbreak occurs is closely linked to three critical factors: the proportion of resources distributed from external sources, the effectiveness of therapeutic resources, and the topological attributes of the underlying contact networks. The accuracy of the dynamical equations was validated by comparison with MC simulations, and the results showed high consistency with the MMC approach. Furthermore, the model reveals how false panic information, resource distribution strategies, and network topology interact to shape epidemic prevalence and thresholds. Simulation results demonstrate several key findings. First, prioritizing the allocation of resources to infected individuals not only effectively suppresses epidemic outbreaks by directly enhancing recovery rates, but also achieves the strongest mitigation of panic-related false information. Second, increasing the total availability of therapeutic resources reduces epidemic prevalence, particularly when resources are scarce. Third, improving the effectiveness of resources further elevates the epidemic threshold and delays outbreak onset. These results jointly suggest that epidemic control strategies should consider not only the total quantity of resources but also their targeted allocation and practical efficiency. This study enriches the theoretical framework of co-evolutionary spreading on multilayer networks and provides valuable practical guidance for epidemic prevention and control. The results emphasize that timely and accurate allocation of medical resources to infected individuals is essential for suppressing epidemics and mitigating the risks associated with panic-related false information. These measures help strengthen the resilience of healthcare systems and improve the efficiency of epidemic response. In future work, we plan to extend the current model by incorporating the dissemination of both true and false information, in order to explore the more complex and competitive interactions between multiple information types and their joint impacts on resource allocation behaviors and epidemic transmission dynamics. This will allow for a more comprehensive understanding of the role of information in shaping epidemic evolution.

## Figures and Tables

**Table 1:** Average relative errors of $\rho^{A}$, $\rho^{R}$, and $\rho^{I}$ obtained from the two methods shown in Figure 4.

Case	$\rho^{A}$ (%)	$\rho^{R}$ (%)	$\rho^{I}$ (%)
Figure 4a	21.51	3.21	1.30
Figure 4b	21.68	7.20	1.74

## Data Availability

All data that support the findings of this study are included within the article.

## References

[1] Buckee C., Noor A., Sattenspiel L. (2021). Thinking clearly about social aspects of infectious disease transmission. Nature.

[2] Riley S., Fraser C., Donnelly C.A., Ghani A.C., Abu-Raddad L.J., Hedley A.J., Leung G.M., Ho L.-M., Lam T.H., Thach T.Q. (2003). Transmission dynamics of the etiological agent of SARS in Hong Kong: Impact of public health interventions. Science.

[3] Flaxman S., Mishra S., Gandy A., Unwin H.J.T., Mellan T.A., Coupland H., Whittaker C., Zhu H., Berah T., Eaton J.W. (2020). Estimating the effects of non-pharmaceutical interventions on COVID-19 in Europe. Nature.

[4] Grassly N.C., Fraser C. (2008). Mathematical models of infectious disease transmission. Nat. Rev. Microbiol..

[5] Xian J., Liu M., Cheng X., Yang M., Xie T., Wang X., Liu M., Zhang Y.-C., Yang D., Sun G.-Q. (2025). Modelling multiscale infectious disease in complex systems. Phys. Rep..

[6] Metcalf C.J.E., Lessler J. (2017). Opportunities and challenges in modeling emerging infectious diseases. Science.

[7] Funk S., Salathé M., Jansen V.A.A. (2010). Modelling the influence of human behaviour on the spread of infectious diseases: A review. J. R. Soc. Interface.

[8] Perra N. (2021). Non-pharmaceutical interventions during the COVID-19 pandemic: A review. Phys. Rep..

[9] Nowzari C., Preciado V.M., Pappas G.J. (2017). Optimal resource allocation for control of networked epidemic models. IEEE Trans. Control Netw. Syst..

[10] Sun Q., Wang Z., Zhao D., Xia C., Perc M. (2022). Diffusion of resources and their impact on epidemic spreading in multilayer networks with simplicial complexes. Chaos Solitons Fractals.

[11] Kermack W.O., McKendrick A.G. (1927). A contribution to the mathematical theory of epidemics. Proc. R. Soc. Lond. A.

[12] Hethcote H.W. (2000). The mathematics of infectious diseases. SIAM Rev..

[13] Huppert A., Katriel G. (2013). Mathematical modelling and prediction in infectious disease epidemiology. Clin. Microbiol. Infect..

[14] Aleta A., Ferraz de Arruda G., Moreno Y. (2020). Data-driven contact structures: From homogeneous mixing to multilayer networks. PLoS Comput. Biol..

[15] Thomas L.J., Huang P., Yin F., Luo X.I., Almquist Z.W., Hipp J.R., Butts C.T. (2020). Spatial heterogeneity can lead to substantial local variations in COVID-19 timing and severity. Proc. Natl. Acad. Sci. USA.

[16] Watts D.J., Strogatz S.H. (1998). Collective dynamics of ‘small-world’ networks. Nature.

[17] Barabási A.L., Albert R. (1999). Emergence of scaling in random networks. Science.

[18] Pastor-Satorras R., Vespignani A. (2001). Epidemic spreading in scale-free networks. Phys. Rev. Lett..

[19] Boccaletti S., Latora V., Moreno Y., Chavez M., Hwang D.-U. (2006). Complex networks: Structure and dynamics. Phys. Rep..

[20] Pastor-Satorras R., Castellano C., Van Mieghem P., Vespignani A. (2015). Epidemic processes in complex networks. Rev. Mod. Phys..

[21] Sun G.-Q., He R., Hou L.-F., Luo X., Gao S., Chang L., Wang Y., Zhang Z.-K. (2025). Optimal control of spatial diseases spreading in networked reaction–diffusion systems. Phys. Rep..

[22] Wang W., Liu Q.-H., Liang J., Hu Y., Zhou T. (2019). Coevolution spreading in complex networks. Phys. Rep..

[23] Saad-Roy C.M., Traulsen A. (2023). Dynamics in a behavioral–epidemiological model for individual adherence to a nonpharmaceutical intervention. Proc. Natl. Acad. Sci. USA.

[24] Wang Z., Li H., Chen J., Hong Z., Yin Q., Xia C. (2024). Coupled propagation dynamics on complex networks: A brief review. Europhys. Lett..

[25] Funk S., Gilad E., Watkins C., Jansen V.A.A. (2009). The spread of awareness and its impact on epidemic outbreaks. Proc. Natl. Acad. Sci. USA.

[26] Zhang Z.-K., Liu C., Zhan X.-X., Lu X., Zhang C.-X., Zhang Y.-C. (2016). Dynamics of information diffusion and its applications on complex networks. Phys. Rep..

[27] Wang Z., Xia C. (2020). Co-evolution spreading of multiple information and epidemics on two-layered networks under the influence of mass media. Nonlinear Dyn..

[28] Bauch C.T., Galvani A.P. (2013). Social factors in epidemiology. Science.

[29] Kivelä M., Arenas A., Barthelemy M., Gleeson J.P., Moreno Y., Porter M.A. (2014). Multilayer networks. J. Complex Netw..

[30] Boccaletti S., Bianconi G., Criado R., del Genio C.I., Gómez-Gardeñes J., Romance M., Sendiña-Nadal I., Wang Z., Zanin M. (2014). The structure and dynamics of multilayer networks. Phys. Rep..

[31] De Domenico M., Granell C., Porter M.A., Arenas A. (2016). The physics of spreading processes in multilayer networks. Nat. Phys..

[32] Arruda G.F., Rodrigues F.A., Moreno Y. (2018). Fundamentals of spreading processes in single and multilayer complex networks. Phys. Rep..

[33] De Domenico M. (2023). More is different in real-world multilayer networks. Nat. Phys..

[34] Bagnoli F., Liò P., Sguanci L. (2007). Risk perception in epidemic modeling. Phys. Rev. E.

[35] Moinet A., Pastor-Satorras R., Barrat A. (2018). Effect of risk perception on epidemic spreading in temporal networks. Phys. Rev. E.

[36] Herrera-Diestra J.L., Meyers L.A. (2019). Local risk perception enhances epidemic control. PLoS ONE.

[37] Granell C., Gómez S., Arenas A. (2013). Dynamical interplay between awareness and epidemic spreading in multiplex networks. Phys. Rev. Lett..

[38] Wang Z., Xia C., Chen Z., Chen G. (2021). Epidemic propagation with positive and negative preventive information in multiplex networks. IEEE Trans. Cybern..

[39] Yin Q., Wang Z., Xia C. (2023). Information–epidemic co–evolution propagation under policy intervention in multiplex networks. Nonlinear Dyn..

[40] Xu J., Li J., Han Z., Zhu P. (2024). Coupled epidemic dynamics with awareness heterogeneity in multiplex networks. Chaos Solitons Fractals.

[41] Zhang Z., Zhu K., Wang F., Liu L., Wang L. (2025). Effects of isolation and information dissemination on epidemic dynamics in multiplex networks. Chaos Solitons Fractals.

[42] Van Wesemael T., Rocha L.E.C., Baetens J.M. (2025). Epidemic risk perception and social interactions lead to awareness cascades on multiplex networks. J. Phys. Complex..

[43] Massaro E., Bagnoli F. (2014). Epidemic spreading and risk perception in multiplex networks: A self-organized percolation method. Phys. Rev. E.

[44] Gao S., Dai X., Wang L., Perra N., Wang Z. (2023). Epidemic spreading in metapopulation networks coupled with awareness propagation. IEEE Trans. Cybern..

[45] Feng M., Liu L., Chen J., Xia C. (2024). Heterogeneous propagation processes between awareness and epidemic on signed multiplex networks. Chaos Solitons Fractals.

[46] An X., Zhang C., Hou L., Wang K. (2024). Coupled epidemic-information propagation with stranding mechanism on multiplex metapopulation networks. IEEE Trans. Comput. Soc. Syst..

[47] Ye Y., Zhang Q., Ruan Z., Cao Z., Xuan Q., Zeng D.D. (2020). Effect of heterogeneous risk perception on information diffusion, behavior change, and disease transmission. Phys. Rev. E.

[48] Zhu X., Liu Y., Wang X., Zhang Y., Liu S., Ma J. (2022). The effect of information-driven resource allocation on the propagation of epidemic with incubation period. Nonlinear Dyn..

[49] Huo L., Yu Y. (2023). The impact of the self-recognition ability and physical quality on coupled negative information-behavior-epidemic dynamics in multiplex networks. Chaos Solitons Fractals.

[50] Han D., Wang X. (2024). Impact of positive and negative information on epidemic spread in a three-layer network. Chaos Solitons Fractals.

